# Surface electromyographic characteristics of forearm muscles after ulnar and radius fracture inchildren

**DOI:** 10.3389/fped.2023.1143047

**Published:** 2023-04-28

**Authors:** Hailing Qiu, Fanling Li, Siqi Zhang, Sheng Xiao, Haobo Liu, Shuangxi Chen, Xin Li, Ke Fang, Jie Wen, Tingzhi Li

**Affiliations:** ^1^ Department of Pediatric Orthopedics, Hunan Provincial People’s Hospital, The First Affiliated Hospital of Hunan Normal University, Changsha, China; ^2^ Department of Anesthesiology, The First People’s Hospital of Chenzhou, Chenzhou, China; ^3^ Department of Anatomy, Hunan Normal University School of Medicine, Changsha, China

**Keywords:** elastic intramedullary nail, children, surface electromyography, ulnar fracture, radiusfracture

## Abstract

**Objective:**

To evaluate the characteristics of forearm muscle activity in children with ulnar and radius fractures during different follow-up periods by surface electromyography.

**Methods:**

A retrospective analysis was performed on 20 children with ulnar and radius fractures treated with an elastic intramedullary nail from October 2020 to December 2021. All children were treated with transcubital casts after surgery. At 2 months and before taking out the elastic intramedullary nail, surface electromyographic signals were collected on the flexor/extension of the wrist and the maximum arbitrary isometric contraction of the grip strength in the forearm flexor and extensor muscles of the forearm. The root-mean-square values and integrated EMG values of the superficial flexor and extensor digitalis of the healthy side and the affected side were collected at the last follow-up and 2 months after surgery, and the co-systolic ratio was calculated. The root-mean-square values and co-systolic ratio were compared and analyzed, and the Mayo wrist function score was evaluated.

**Results:**

The mean follow-up time was (8.4 ± 2.85) months. Mayo scores were (87.42 ± 13.01) and (97.69 ± 4.50) points at the last follow-up and two months after surgery, respectively (*p* < 0.05). In the test of grip strength, 2 months after surgery, the grip strength of the affected side was lower than that of the healthy side (*p* < 0.05), and the maximum and mean values of the superficial flexor of the affected side were lower than those of the healthy side (*p* < 0.05). At the last follow-up, there was no difference in the grip strength between the affected side and the healthy side (*p* > 0.05), and no difference in the maximum RMS, mean RMS and cooperative contraction ratio of the superficial flexor and digital extensor muscles between the affected side and the healthy side (*p* > 0.05).

**Conclusion:**

Satisfactory results can be obtained after elastic intramedullary napping in children with ulnar and radius fractures. However, 2 months after surgery, the grip strength of the affected side is small, and the electrical activity of the forearm muscle is low during flexion and extension activities of the wrist joint, which has not returned to normal, suggesting that children orthopaedic clinicians should remind children to conduct timely and effective rehabilitation training after the removal of the cast.

## Introduction

Fracture is the most common type of accidental injury in children, and ulna radius fracture is the most common type of fracture in children, accounting for 17.8%–40% of all fractures in children (1, 2). Most ulna and radius fractures are usually treated successfully with a tubular cast after manual reduction, and surgical treatment is required when the fracture is unstable or cannot be manually reduced (3). Elastic intramedullary nail fixation has become the gold standard surgical method for treating children with both ulnar and radius fractures due to its characteristics of a small incision, short operation time, fast healing speed, and low cost (4). However, the physiological and anatomical structure of the ulna and radius in children is special, and the forearm interosseous membrane and muscle tissue are easily damaged, which is not conducive to postoperative fracture rehabilitation. Successful treatment of ulnar and radius fractures should not only achieve fracture healing and joint activity recovery but also achieve the recovery of muscle strength, such as grip strength (5), which is not only related to the treatment method but also involves whether the children's early activity/rehabilitation training is in place.

Children with ulnar and radius fractures who receive elastic intramedullary nailing and internal fixation usually need a tubular cast for 4–6 weeks. Complications such as muscle atrophy, functional limitations, and joint stiffness may result from prolonged cast immobilization (6, 7). Although these symptoms usually return to normal a few weeks after the cast is removed, it is a painful and lengthy recovery process for children. Due to the relatively young age of the children, the trauma pain after the injury and surgery is fresh in their memory, so the children are very afraid of the pain during the postoperative rehabilitation, which seriously affects their compliance with functional exercise. Moreover, the bones are still fragile due to the loss of bone mass after removing the cast, so some doctors may suggest that children avoid sports within 4–6 weeks after cast removed to prevent bone breakage again (8). The excessive protection of some parents undoubtedly increases the psychological protection of children on the affected limb. Avoiding daily activities with the affected limb is not conducive to the recovery of joint activities and muscle strength of the affected limb, nor does it meet the goal of accelerated rehabilitation surgery, affecting the daily life and learning of children.

Surface electromyography (sEMG) measures the time signal of the bioelectrical activity of the neuromuscular system recorded from the muscle surface and is the gold standard for analyzing muscle activation (9). In recent years, sEMG has been applied in advanced prosthetic control, gesture recognition, rehabilitation, biomechanics and other fields (10, 11) and has been widely used in the quantitative measurement of muscle activity in patients with cerebral palsy (12, 13), Duchenne muscular dystrophy (14), stroke (15, 16) and other upper limb movement disorders. Studies have shown that sEMG can distinguish between pathological and normal muscle activation, effectively evaluate upper limb function and therapeutic effect, and is a common method for studying upper limb muscle load (17–20). In these studies, the amplitude of surface electromyographic signals was used to provide quantitative measurements, in which the root-mean-square value (RMS), as an important time-domain index of sEMG, was used to describe the average variation characteristics of electromyography over a period, which was related to the supplement of motor units and the synchronization of excitatory rhythms. Integrated electromyography (iEMG) can be used as an indirect supplementary measure of motor units and is often used to examine muscle activity for various tasks. The Synergistic shrinkage ratio reflects the proportion of antagonistic muscles during active contraction and the coordination between active and antagonistic muscles during joint movement. Proper coordination of active and antagonistic muscles contributes to joint stability and motor coordination (11).

Most studies on children with ulnar and radius fractures assess the therapeutic effect and functional results of fractures from the clinical manifestations and imaging aspects (21–23). There are few reports on the recovery of muscle activity after upper limb fracture surgery in normal children, and whether the postoperative rehabilitation training of children is timely and effective is unknown. The purpose of this study is to evaluate the muscle activity status of children after fracture of the radius and ulna by surface electromyography. These results will help pediatric orthopedic surgeons understand the micro-recovery of muscle activity after children's fractures and provide a reference for clinical rehabilitation.

## Materials and methods

The subjects of this study were selected from 20 children with ulnar and radius fractures hospitalized in the pediatric orthopedic Department of Hunan provincial people's hospital from October 2020 to December 2021, including 15 males and 5 females aged from 5 to 14 years old, with an average age of (7.90 ± 2.43) years old and an average height of (131.38 ± 17.43) cm. The average weight was 31.02 ± 11.4 kg, and the BMI was 17.39 ± 2.81. There were 18 right-handedness and 2 left-handedness. One of them changed from right-handedness to left-handedness after surgery. 10 on the left, 10 on the right; Fractures were located in the upper 1/3 of the ulna radius in 6 patients and the lower 2/3 in 14 patients. There were 12 patients with open reduction and 8 patients with closed reduction. This study was reviewed and approved by the hospital's Ethics committee, and all children's families were informed and signed informed consent.

**Inclusion criteria:** (1) Patients diagnosed as fracture of ulnar and radius fixed with an elastic intramedullary nail; (2) age below 14 years old; (3) no strenuous exercise 48 h before EMG test; (4) the follow up data were complete.

**Exclusion criteria**: (1) Diagnosed as multiple fractures; (2) chronic fracture; (3) pathological fracture caused by infectious diseases or metabolism disease; (4) pathological fracture with other bone or neuromuscular diseases and bone tumours; Intellectual disability, inability to follow instructions to complete prescribed actions; (5) combined with nerve or vascular injury.

All the children were treated with elastic intramedullary nail fixation under general anaesthesia, and were fixed with long arm cast for 4–6 weeks after surgery. After surgery, routine finger function exercises and motion contraction exercises of forearm muscle groups were performed after surgery. x-ray films were reviewed 3 days, 1 month and 2 months after surgery, and x-ray films showed that the plaster was removed after fracture healing. After discharge, the medical staff supervised the children's home rehabilitation training on a daily basis via telephone.

### Observation and evaluation indicators

#### Mayo wrist function score

During regular follow-up after plaster removal, the wrist function of children with Mayo wrist function score was used, with a total score of 100, including 25 points for pain, 25 points for functional status, 25 points for a range of motion and 25 points for grip strength. Scores <60 is poor, 60–79 is fair, 80–89 is good, 90–100 is excellent. Excellent and good rate = (excellent cases + good cases)/total cases × 100%.

#### Grip strength test

The child sat in a straight-backed chair with feet flat on the floor, elbows bent 90° on a table, forearms in a neutral position, wrists extended in a neutral position and held a calibrated electronic grip strength tester with adjustable grip handles (CAMRY, model EH101, MAX:90 Kg, *d* = 100 g) for maximum strength grasping, testing 3 times, each duration of 5–7 s, each time interval of 1 min, take the maximum reading for analysis. As some of the children had not used the gripometer before the test, the evaluator conducted a demonstration before the test and asked the children to gently hold the gripometer, observe the value changes of the electronic display screen, and experience the test process. During the grip strength test, the surface electromyographic signals of forearm flexors and forearm extensors were collected synchronically. The surface electromyographic signals of the maximum grip strength were analyzed. The analysis software of the surface electromyographic test system was used to calculate the maximum RMS, the mean value of RMS and the mean value of iEMG. The Synergistic shrinkage ratio was calculated according to iEMG = iEMG for the superficial flexor /(iEMG for extensor + iEMG for superficial flexor).

#### Surface electromyography test

sEMG tests were performed on the upper forearm muscle groups of the children 2 months and before elastic intramedullary nailing. The test results for 2 months and the last time after surgery were analyzed. The test and evaluation will be conducted by personnel. The test was conducted in a surface electromyography room at 26 ± 2°C using the Flexcomp surface electromyography Test System (THOUGHT TECHNOLOGY, Canada). The test electrode was a disposable Triodes dry electrode, round, with a peripheral diameter of 5.6 cm and an electrode diameter of 1.0 cm. The distance between the recording electrode and the reference electrode centre is 2 cm. Subjects were fully exposed to both upper limbs, and the skin was degreased with 75% medical alcohol. The electrodes were placed on the abdomen of the fingers' superficial flexor and extensor muscles on both sides and were parallel to the long axis of the muscle fibres. Collect maximum isometric contraction at random (maximum voluntary isometric contraction MVIC) wristwatch recorded during flexion and extension. The process of test was explained and demonstrated by a special stuff to patient before the test. The mean value of RMS, maximum value of RMS, and iEMG value are processed by the data processing system of the device.

During the wrist flexion and extension test, the child was seated, with elbow flexion of 90° and forearm pronation, followed by the metronome 3 s/time to complete wrist flexion and extension, requiring flexion and extension to the maximum range of motion, 20 times; In order to eliminate unnecessary interference of the upper limbs at the beginning of the movement, the waveforms of the first 2 flexions and the last 1 flexion and extension were eliminated, the mean value of RMS and iEMG value were analyzed, and the synergistic shrinkage ratio was calculated. During the grip strength test, the surface electromyographic signals of the superficial flexor and extensor fingers were evaluated simultaneously. For details, see Grip Strength test.

### Statistical analysis

All data were recorded in Excel tables, and the results were expressed as (*x* ± *s*). Statistical software SPSS21.0 was used to process the data. The maximum and mean values of RMS in the finger's superficial flexor and extensor muscles on the affected and healthy sides were compared during the same follow-up period. The mean values of RMS, maximum values of RMS in the muscle of the same name and the Synergistic shrinkage ratio of the muscle on the same side were compared 2 months after surgery and at the last follow-up. As some data did not conform to normal distribution, Wilcoxon rank-sum test was used. The correlation between the maximum grip strength and the maximum RMS of the superficial flexor and extensor of the finger in the same period was analyzed. *P* < 0.05 was considered statistically different.

## Results

The average follow-up of all the children was (8.4 ± 2.85) months. The Mayo score was (87.42 ± 13.01) two months after surgery, and the good and good rate was 85%. At the last follow-up, the Mayo score of the wrist was significantly increased (97.69 ± 4.50), and the good and good rate was 100%. Compared with the follow-up 2 months after surgery, there was a statistically significant difference in the wrist Mayo score at the last follow-up (*p* < 0.05).

Table 1 shows that all the children underwent a grip strength test, and the grip strength of the affected side was lower than that of the healthy side at the follow-up 2 months after surgery, with statistical significance (*p* < 0.05). At the last follow-up, there was no difference in grip strength between the affected and healthy sides (*p* > 0.05).

**Table 1:** The results of grip strength (kg) on the affected side and the healthy side, the maximum value of electromyographic RMS (µV), the mean value of RMS (µV), and the synergistic shrinkage ratio of the superficial flexor and digital extensor muscles during gripping with the grip meter 2 months after the operation and the last follow-up.

	Follow up 2 months after surgery		Last follow-up	
	Affected side	Healthy side	Affected side	Healthy side
Grip strength	9.28 ± 5.41*	12.36 ± 6.49	10.88 ± 6.54**	11.48 ± 6.20**
Superficial flexor digitalis Max	267.71 ± 114.65*	321.91 ± 132.28	304.38 ± 119.24**	334.35 ± 144.19
Extensor digitalis Max	305.65 ± 128.83*	398.22 ± 135.47	52.80 ± 134.07**	3,411.34 ± 152.02
Superficial flexor digitalis mean	164.64 ± 69.24*	183.41 ± 65.48	183.46 ± 70.19**	188.84 ± 69.06
Extensor digitalis mean	190.50 ± 81.26*	237.28 ± 78.82	217.18 ± 88.99**	244.29 ± 84.39
Synergistic shrinkage ratio	0.46 ± 0.11	0.44 ± 0.10	0.46 ± 0.11	0.44 ± 0.10

* Means the comparison between the affected side and the healthy side in the same follow-up period, *p* < 0.05.

** Compared with the same side at the last follow-up 2 months after surgery, *p* < 0.05.

At the last follow-up, the grip strength of the affected side was increased (*p* < 0.05), while the grip strength of the healthy side was decreased (*p* < 0.05). In the surface electromyography test conducted simultaneously with the grip strength test, the maximum value of RMS of the superficial flexor of the finger, the maximum value of RMS of the extensor of the finger, the mean value of the superficial flexor of the finger and the mean value of the extensor of the finger on the affected side were significantly lower than those on the healthy side 2 months after surgery (*p* < 0.05). At the last follow-up, there was no statistical difference between the healthy and affected sides (*p* < 0.05). Compared with 2 months after surgery, the maximum value of RMS of the superficial flexor and extensor of the finger and the mean value of RMS of the superficial flexor and extensor of the finger were increased at the last follow-up (*p* < 0.05).

Table 2 shows that at the last follow-up, there was a positive correlation between the grip strength of the affected side and the maximum value of the superficial flexor finger on the affected side (*r* = 0.469, *p* = 0.037). There was a positive correlation between the grip strength of the healthy side and the maximum value of the finger's flexor on the healthy side (*r* = 0.452, *p* = 0.045). There was no linear correlation between grip strength and the maximum value of the superficial flexor of the finger at 2 months after the operation.

**Table 2:** Pearson correlation coefficient results of the correlation between maximum grip strength and maximum RMS of superficial digital flexor muscle.

	Follow up 2 months after surgery		Last follow-up	
	Affected side	Healthy side	Affected side	Healthy side
Gripstrength	0.398	0.334	0.469*	0.452*

* Indicates *p* < 0.05.

Table 3 shows that when all the children underwent maximum voluntary flexion and extension of the wrist, the mean value of the RMS of the superficial flexor and extensor digitalis on the affected side was smaller than that on the healthy side 2 months after surgery and the difference was statistically significant (*p* < 0.05). Compared with 2 months after surgery, the mean value of RMS of the flexor and extensor of the finger on the affected side was significantly increased (*p* < 0.05). The mean value of the extensor digitalis of the healthy side decreased, and the difference was statistically significant (*p* < 0.05).

**Table 3:** The mean value (µV) of surface electromyographic signal (RMS) and synergistic shrinkage ratioof the superficial flexor and digital extensor muscles of the affected and healthy sides during maximum voluntary flexion and extension of the wrist in children with ulnar radius fracture at 2months and the last follow-up.

	Follow up 2 months after surgery		Last follow-up	
	Affected side	Healthy side	Affected side	Healthy side
Grip strength	62.47 ± 31.42*	76.99 ± 36.35	78.79 ± 38.99**	74.70 ± 36.35
Superficial flexor digitalis Max	66.08 ± 28.05*	83.36 ± 29.34	81.31 ± 33.04**	81.84 ± 28.70**
Synergistic shrinkage ratio	0.48 ± 0.09	0.47 ± 0.10	0.49 ± 0.10	0.47 ± 0.10

* Means the comparison between the affected side and the healthy side in the same follow-up period, *p* < 0.05.

** Compared with the same side at the last follow-up 2 months after surgery, *p* < 0.05.

## Discussion

This paper discusses the adverse effects of disuse muscular atrophy caused by immobilization on the macroscopical and microscopical muscles, mainly manifested as decreased muscle endurance and strength and decreased muscle fibre area and volume. Animal experimental studies found that when the sciatic nerve of the left hind limb of 10-week-old mice was incised and fixed with plaster, the bone strength and cross-sectional area of the rectus femoris muscle of the experimental limb decreased by about 10% and 30%, respectively 4 weeks after operation compared with the intact limb (24). Other animal experiments also found histological evidence of muscle atrophy in the plaster-fixed limb (25). In the absence of motor nerve injury, after 10 days of human lower limb plaster fixation, quadriceps cross-sectional area and maximum leg extension strength in one repeat decreased by 11.8% and 41.6%, respectively (26). Armangil et al. (27) found that after the operation of a volar plate for distal radius fracture, the pronator strength and endurance of the affected side lost 18.5% and 12.9% on average compared with that of the healthy side. The affected side's grip strength was also smaller than that of the healthy side in the weeks prior to distal radius fracture in elderly patients (28). In this study, similar to the above results, the grip strength of the affected side was still significantly lower than that of the healthy side at the short-term follow-up 2 months after surgery. Some studies have shown that the grip strength and hand muscle working ability of patients with closed radial fracture were significantly decreased in the 4th week after surgery but significantly improved in the 6th and 8th week after surgery (5), but such results did not seem to occur in the children in this study at the 8th week after surgery, suggesting from the side that the muscle strength of the affected forearm did not return to normal at this time. After a mean follow-up of 8.4 months, the grip strength of the affected side increased, the grip strength of the healthy side decreased, and finally the grip strength of both sides was equivalent, which was consistent with the expected goal of elastic intramedullary nailing.

Activation of the forearm muscles is important for producing stable grip strength (29), and grip strength are generated by the forearm's flexors (30). Mobach et al. (31) found that 6 weeks of hand fixation after distal radius fracture in adults resulted in a significant reduction in the amplitude and area of the hand's inherent complex muscle action potential. In this study, the peak value and mean value of the electromyographic activity amplitude of the flexor digitalis superficial and extensor digitalis of the forearm on the affected side were significantly lower than those on the healthy side during the grip strength test 2 months after surgery. Although an important factor in the selection of elastic intramedullary nail therapy was due to the small trauma and easy early recovery, the electromyographic activity of the affected forearm muscle was significantly less than that of thehealthy side, indicating that the electromyographic activity of the affected forearm muscle did not return to normal 2 months after surgery, especially in the superficial flexor muscle, which to some extent also reflected the consistency of grip strength and electromyographic activity results. We speculated that the reason for this result might be that although the plaster had been removed for a considerable period two months after surgery, combined with the excellent and good rate of 85% of the wrist Mayo score, the wrist joint activity returned to normal, but the early rehabilitation training was not in place due to pain, joint stiffness, overprotection and other physiological and psychological factors. The muscle mass and strength of the affected limb have not been fully recovered, leading to incomplete activation of the muscle motor unit and little muscle strength in children when they are active. In this study, one child relied too much on the healthy side limb, resulting in changes in the dominant side, which supported the above views. As with all surgical procedures, patient compliance is crucial in forearm fractures (32). Psychological exercises can positively impact the outcome of distal radius fractures requiring immobilization (33), and targeted psychological counselling can be performed at follow-up if necessary. Studies have found that muscle hypertrophy occurs in the first 5 weeks after immobilization, and more than 50% of hypertrophy during rehabilitation also occurs in the first 5 weeks of rehabilitation (34). In this study, at the follow-up 2 months after surgery, the child was in the first 5 weeks of rehabilitation, but the muscle activity of the affected limb did not return to normal at this time, suggesting that timely intervention and effective rehabilitation training should be conducted after the removal of the plaster.

In adults, fixation after fracture leads to muscle expense atrophy and rapid loss of muscle mass and strength, which can be offset by retraining and eventually complete recovery (28, 35). This phenomenon also occurs in children. As time passed, the affected side's upper limb gradually returned to normal activities. By 8.4 months after surgery, the Mayo Mayo score rate of the wrist was 100%, and the functional status, range of motion, and grip strength of the wrist on the affected side had returned to normal. The electromyographic activities of the flexor and extensor of the finger of the affected side were significantly increased compared with the follow-up of 2 months. The electromyographic activities of the muscles of the affected side and the healthy side were symmetrical. The changes in grip strength were consistent with those of the affected side. However, most children will have the elastic intramedullary nailing device removed at this time. Although it is also a minimally invasive operation, the second operation is another challenge for children who have not fully recovered physically and psychologically. We should ensure that the child is prepared for a smooth recovery before removing internal fixation.

Some scholars have used surface electromyography to study the relationship between grip strength and forearm muscle activity. Hoozemans et al. (36) used surface electromyography to predict the grip strength of 6 forearm muscles, including superficial flexor digitalis and extensor digitalis, in 8 healthy male subjects with the right hand. The results showed little absolute difference between observed and predicted grip strength and suggested that grip strength could be effectively predicted based on the EMG of three of the six forearm muscles. Duque et al. (37) studied the relationship between grip strength on the dynamometer and standard electromyography of the superficial flexor of the finger in 20 subjects using static calibration methods, and the results showed that the correlation coefficient between predicted grip strength and observed grip strength was 0.895. Gurram et al. (38) used power curve regression analysis to study the relationship between the EMG of finger flexors and grip strength under different static and dynamic loads, and the results showed that the correlation coefficient between the two was 0.91–0.99 under static loads and 0.78–0.99 under dynamic loads. Takala et al. (20) conducted surface EMG tests on the fingers' right extensor and superficial flexor muscles of 11 healthy right-handed adults during gripping, pushing and lifting activities and showed the highest correlation coefficient between external load and EMG activity was 0.25–0.66. The results of this study were similar to those of Takala et al. (20). Through the correlation analysis of the maximum grip strength and the maximum RMS of the superficial flexor of the finger, it was found that the grip strength of the affected side and the healthy side were moderately correlated with the maximum RMS of the superficial flexor of the finger at the last follow-up, and the correlation coefficient ranged from 0.452 to 0.469. It is suggested that the maximum RMS of the superficial flexor muscle may reflect grip strength to some extent. However, compared with other results, the correlation coefficient in this study was significantly lower, which may be related to the age of test subjects, test posture, electrode position, muscle size and grip gauge width. Gonzalez et al. (39) also pointed out that the performance of normally developing children in experiments related to forearm muscle contraction ability was age-related. Secondly, compared with the adults in the above study, although the target muscles are the superficial flexor and digital extensor muscles, the children's muscle development is not mature, the cross-sectional muscle area and muscle fibre size are significantly smaller, and the electromyographic crosstalk collected by the surface electromyographic electrode is relatively more obvious than that of adults, which may affect the results of the study. In addition, the width of the gripometer is larger than that of young children's palms, which also affects muscle activity to some extent, which may lead to a low correlation.

In this study, through the analysis of the electromyographic activity during flexion and extension of the wrist joint, the mean electromyographic activity RMS of the superficial flexor and extensor of the finger on the affected side did not recover to the level of the healthy side 2 months after surgery. After an average follow-up of 8.4 months, the electromyographic activity of the superficial flexor and extensor of the finger on the affected side increased significantly, while the electromyographic activity of the extensor of the finger on the healthy side decreased, and thebilateral electromyographic activity returned to basic symmetry. Similar to the changes in the electromyographic activity of the superficial flexor and extensor of the finger in the grip strength test, it again suggested that the electromyographic activity of the forearm muscles of the children did not return to normal during the flexion and extension of the grip and wrist joints in the early rehabilitation 2 months after surgery. Therefore, pediatric orthopedic clinicians should remind children to conduct timely and effective rehabilitation training after removing the cast. Rahmati et al. indicate that neuromuscular electrical stimulation (NMES) has been used as a safe training strategy in increasing skeletal muscle strength and mass in healthy young and older adults (40). Others indicated that aerobic, resistance and combined exercise training are also other type of rehabilitation training programs in reducing discomfort and increasing the quality of life in patients (41).

Studies have shown that the wrist flexors are highly task-dependent and contribute the most to volar-dominated tasks but show very low muscle activity in dorsal-dominant tasks and play a major role in generating grip strength and wrist flexion. In almost all cases, the wrist extensor is at a medium-high level, showing greater activity than the wrist flexor during hand dominance tasks to resist task-dependent changes, and plays a major role in wrist stabilization in countering the forces generated by the wrist flexors (42, 43). In this study, the electromyographic activities of the superficial flexors and extensors of the middle finger were consistent with the above conclusions. In addition, compared with other populations, such as cerebral palsy (11), the test subjects in this study were all children with normal development. No matter in the grip strength test or wrist flexion and extension activities, the cooperative contraction ratio of the affected side showed no difference from that of the healthy side, which also suggested that although the electromyographic activity of the superficial flexor and extensor of the affected side was less 2 months after surgery, the coordinated contraction ability between the two was not affected. Motor efficiency and hand function were not limited.

The limitations of this study are as follows: First, the sample size is small. We only studied the surface EMG features of the forearm muscle groups of the injured limb and the healthy limb in children with ulna and radius fractures and did not make a comparative analysis with the surface EMG features of the upper limb muscles of the normal children without injuries. However, since the subjects in this study were normally developing children, it was assumed that their muscle development and muscle activity were normal control group children, and the surface electromyographic signals on the healthy and the affected side after recovery were normal. Secondly, we did not thoroughly investigate whether the children's lifestyle and the psychological state changed after surgery, which will affect the effect of rehabilitation training. Third, the forearm muscle thickness was not measured in this study, which may partly influence the evaluation of EMG signals on the surface of the forearm muscles.

## Conclusion

In summary, the elastic intramedullary nail treatment in children with ulnar and radius fractures can finally achieve satisfactory results. However, 2 months after surgery, the grip strength of the affected side is small, and the electrical activity of the forearm muscle is low during the flexion and extension activities of the wrist joint, which has not returned to normal, suggesting that children orthopaedic clinicians should remind children to conduct timely and effective rehabilitation training after the removal of the cast.

## Data Availability

The original contributions presented in the study are included in the article, further inquiries can be directed to the corresponding author.

## References

[1] Naranje SM Erali RA Warner WC Sawyer JR Kelly DM. Epidemiology of pediatric fractures presenting to emergency departments in the United States. J Pediatr Orthop. (2016) 36(4):e45–8. 10.1097/BPO.0000000000000595 26177059

[2] Bell SW McLaughlin D Huntley JS. Paediatric forearm fractures in the west of Scotland. Scott Med J. (2012) 57(3):139–43. 10.1258/smj.2012.012018 22859804

[3] Griffet J el Hayek T Baby M. Intramedullary nailing of forearm fractures in children. J Pediatr Orthop B. (1999) 8(2):88–9. PMID: 10218166

[4] Korhonen L Lutz N Sinikumpu JJ. The association of metal frame construct of ESIN and radiographic bone healing of pediatric forearm fractures. Injury. (2020) 51(4):856–62. 10.1016/j.injury.2020.03.028 32184011

[5] Solanki PV Mulgaonkar KP Rao SA. Effect of early mobilisation on grip strength, pinch strength and work of hand muscles in cases of closed diaphyseal fracture radius-ulna treated with dynamic compression plating. J Postgrad Med. (2000) 46(2):84–7. PMID: 11015774

[6] Ceroni D Martin X Delhumeau-Cartier C Rizzoli R Kaelin A Farpour-Lambert N. Is bone mineral mass truly decreased in teenagers with a frst episode of forearm fracture? A prospective longitudinal study. J Pediatr Orthop. (2012) 32(6):579–86. 10.1097/BPO.0b013e31824b2b1f 22892619

[7] Boero S Michelis MB Calevo MG Stella M. Multiple forearm diaphyseal fracture: reduction and plaster cast control at the end of growth. Int Orthop. (2007) 31(6):807–10. 10.1007/s00264-006-0255-z 17109178 PMC2266673

[8] Caruso G Caldari E Sturla FD Caldaria A Re DL Pagetti P Management of pediatric forearm fractures: what is the best therapeutic choice? A narrative review of the literature. Musculoskelet Surg. (2021) 105(3):225–34. 10.1007/s12306-020-00684-6 33058085 PMC8578082

[9] Gazzoni M Celadon N Mastrapasqua D Paleari M Margaria V Ariano P. Quantifying forearm muscle activity during wrist and finger movements by means of multi-channel electromyography. PLoS One. (2014) 9(10):e109943. 10.1371/journal.pone.0109943 25289669 PMC4188712

[10] Pradhan A He J Jiang N. Multi-day dataset of forearm and wrist electromyogram for hand gesture recognition and biometrics. Sci Data. (2022) 9(1):733. 10.1038/s41597-022-01836-y 36450807 PMC9712490

[11] Jarque-Bou NJ Sancho-Bru JL Vergara M. A systematic review of EMG applications for the characterization of forearm and hand muscle activity during activities of daily living: results challenges, and open issues. Sensors (Basel). (2021) 21(9):3035. 10.3390/s21093035 33925928 PMC8123433

[12] Sarcher A Raison M Ballaz L Lemay M Leboeuf F Trudel K Impact of muscle activation on ranges of motion during active elbow movement in children with spastic hemiplegic cerebral palsy. Clin Biomech (Bristol, Avon). (2015) 30(1):86–94. 10.1016/j.clinbiomech.2014.10.009 25467763

[13] Xu K Mai J He L Yan X Chen Y. Surface electromyography of wrist flexors and extensors in children with hemiplegic cerebral palsy. PM R. (2015) 7(3):270–5. 10.1016/j.pmrj.2014.09.009 25244997

[14] Janssen MM Harlaar J de Groot IJ. Surface EMG to assess arm function in boys with DMD: a pilot study. J Electromyogr Kinesiol. (2015) 25(2):323–8. 10.1016/j.jelekin.2015.01.008 25701200

[15] Vinstrup J Calatayud J Jakobsen MD Sundstrup E Jørgensen JR Casaña J Hand strengthening exercises in chronic stroke patients: dose-response evaluation using electromyography. J Hand Ther. (2018) 31(1):111–21. 10.1016/j.jht.2017.01.004 28527751

[16] Kimura N Sato M Kobayashi Y Naito E. Augmented activity of the forearm extensor muscles induced by vibratory stimulation of the palm of the hand in individuals with subacute post-stroke hemiplegia. Brain Inj. (2022) 36(6):782–91. 10.1080/02699052.2022.2048694 35430945

[17] Nadzri AA A Ahmad SA Marhaban MH Jaafar H. Characterization of surface electromyography using time domain features for determining hand motion and stages of contraction. Australas Phys Eng Sci Med. (2014) 37:133–7. 10.1007/s13246-014-0243-3 24443218

[18] Sanger TD. Use of surface electromyography (EMG) in the diagnosis of childhood hypertonia: a pilot study. J Child Neurol. (2008) 23:644–8. 10.1177/0883073807313045 18344454 PMC2837764

[19] Grunt S Henneman WJ Bakker MJ Harlaar J van der Ouwerkerk WJ van Schie P Effect of selective dorsal rhizotomy on gait in children with bilateral spastic paresis: kinematic and EMG-pattern changes. Neuropediatrics. (2010) 41(5):209–16. 10.1055/s-0030-1267983 21210336

[20] Takala EP Toivonen R. Placement of forearm surface EMG electrodes in the assessment of hand loading in manual tasks. Ergonomics. (2013) 56(7):1159–66. 10.1080/00140139.2013.799235 23713662

[21] Sen RK Tripathy SK Kumar S Aggarwal S Tamuk T. Ipsilateral proximal and distal forearm fracture/fracture dislocation in children. J Pediatr Orthop B. (2011) 20(3):129–37. 10.1097/BPB.0b013e328341df92 21127435

[22] Colaris JW Allema JH Biter LU Reijman M van de Ven CP de Vries MR Conversion to below-elbow cast after 3 weeks is safe for diaphyseal both-bone forearm fractures in children. Acta Orthop. (2013) 84(5):489–94. 10.3109/17453674.2013.850010 24171685 PMC3822135

[23] Roeder C Alves C Balslev-Clausen A Canavese F Gercek E Kassai T Pilot study and preliminary results of biodegradable intramedullary nailing of forearm fractures in children. Children (Basel). (2022) 9(5):754. 10.3390/children9050754 35626931 PMC9140014

[24] Minematsu A Imagita H Kanemura N Yoshimura O. The progression of bone and muscle atrophy in mice hind limb with immobilization. Hiroshima J Med Sci. (2006) 55(3):79–83. PMID: 16995493

[25] Ishikawa T Shimizu M Mikawa Y Zhu BL Quan L Li DR Pathology of experimental disuse muscular atrophy in rats. Connect Tissue Res. (2005) 46(2):101–6. 10.1080/03008200590954131 16019420

[26] Thom JM Thompson MW Ruell PA Bryant GJ Fonda JS Harmer AR Effect of 10-day cast immobilization on sarcoplasmic reticulum calcium regulation in humans. Acta Physiol Scand. (2001) 172(2):141–7. 10.1046/j.1365-201X.2001.00853.x 11442454

[27] Armangil M Bezirgan U Başarır K Bilen G Demirtaş M Bilgin SS. The pronator quadratus muscle after plating of distal radius fractures: is the muscle still working? Eur J Orthop Surg Traumatol. (2014) 24(3):335–9. 10.1007/s00590-013-1193-2 23435787

[28] Szulc P. Impact of bone fracture on muscle strength and physical performance-narrative review. Curr Osteoporos Rep. (2020) 18(6):633–45. 10.1007/s11914-020-00623-1 33030682

[29] Mukaiyama K Irie K Takeda M Yamashita R Uemura S Kanazawa S Load distribution and forearm muscle activity during cylinder grip at various grip strength values. Hand Surg Rehabil. (2022) 41(2):176–82. 10.1016/j.hansur.2021.12.010 35074561

[30] Hong SW Kang JH Kim JS Gong HS. Association between forearm cortical bone properties and handgrip strength in women with distal radius fractures: a cross-sectional study. PLoS One. (2020) 15(12):e0243294. 10.1371/journal.pone.0243294 33270744 PMC7714147

[31] Mobach T Brooks J Breiner A Warman-Chardon J Papp S Gammon B Impact of disuse muscular atrophy on the compound muscle action potential. Muscle Nerve. (2020) 61(1):58–62. 10.1002/mus.26730 31588576

[32] Kuyucu E Koçyiğit F Ciftçi L. The importance of patient compliance in nonunion of forearm fracture. Int J Surg Case Rep. (2014) 5(9):598–600. 10.1016/j.ijscr.2014.04.033 25105774 PMC4200879

[33] Einsiedel T Herzig D Grön G Mayer J Becker C Gebhard F. Mental practice has influence on limitation of motion and muscle atrophy following immobilisation of the radiocarpal joint—a prospective randomised experimental study. Z Orthop Unfall. (2011) 149(3):288–95. 10.1055/s-0030-1270918 21534184

[34] Stevens JE Walter GA Okereke E Scarborough MT Esterhai JL George SZ Muscle adaptations with immobilization and rehabilitation after ankle fracture. Med Sci Sports Exerc. (2004) 36(10):1695–701. 10.1249/01.MSS.0000142407.25188.05 15595289

[35] Karlsson M Nilsson JA Sernbo I Redlund-Johnell I Johnell O Obrant KJ. Changes of bone mineral mass and soft tissue composition after hip fracture. Bone. (1996) 18(1):19–22. 10.1016/8756-3282(95)00422-X 8717532

[36] Hoozemans MJ van Dieën JH. Prediction of handgrip forces using surface EMG of forearm muscles. J Electromyogr Kinesiol. (2005) 15(4):358–66. 10.1016/j.jelekin.2004.09.001 15811606

[37] Duque J Masset D Malchaire J. Evaluation of handgrip force from EMG measurements. Appl Ergon. (1995) 26(1):61–6. 10.1016/0003-6870(94)00003-H 15677002

[38] Gurram R Rakheja S Gouw GJ. A study of hand grip pressure distribution and EMG of finger flexor muscles under dynamic loads. Ergonomics. (1995) 38(4):684–99. 10.1080/00140139508925140 7729396

[39] Gonzalez M Su H Fu Q. Age-dependent upper limb myoelectric control capability in typically developing children. IEEE Trans Neural Syst Rehabil Eng. (2022) 30:1009–18. 10.1109/TNSRE.2022.3166800 35412985

[40] Rahmati M Gondin J Malakoutinia F. Effects of neuromuscular electrical stimulation on quadriceps muscle strength and mass in healthy young and older adults: a scoping review. Phys Ther. (2021) 101(9):pzab144. 10.1093/ptj/pzab144 34106246

[41] Rahmati M Malakoutinia F. Aerobic, resistance and combined exercise training for patients with amyotrophic lateral sclerosis: a systematic review and meta-analysis. Physiotherapy. (2021) 113:12–28. 10.1016/j.physio.2021.04.005 34555670

[42] Forman DA Forman GN Robathan J Holmes MWR. The influence of simultaneous handgrip and wrist force on forearm muscle activity. J Electromyogr Kinesiol. (2019) 45:53–60. 10.1016/j.jelekin.2019.02.004 30822679

[43] Forman DA Forman GN Holmes MWR. Wrist extensor muscle activity is less task-dependent than wrist flexor muscle activity while simultaneously performing moderate-to-high handgrip and wrist forces. Ergonomics. (2021) 64(12):1595–605. 10.1080/00140139.2021.1934564 34024262

